# The value of the sagittal-oblique MRI technique for injuries of the anterior cruciate ligament in the knee

**DOI:** 10.2478/raon-2013-0006

**Published:** 2013-02-01

**Authors:** Dragoslav Nenezic, Igor Kocijancic

**Affiliations:** 1 Clinical Centre of Montenegro, Podgorica, Montenegro; 2 Institute of Radiology. University Medical Centre, Ljubljana, Slovenia

**Keywords:** knee MRI, anterior cruciate ligament, partial rupture, partial tears, sagittal-oblique technique

## Abstract

**Background:**

Complete rupture of the anterior cruciate ligament (ACL) does not represent a diagnostic problem for the standard magnetic resonance (MR) protocol of the knee. Lower accuracy of the standard MR protocol for partial rupture of the ACL can be improved by using additional, dedicated MR techniques. The study goal was to draw a comparison between sagittal-oblique MR technique of ACL imaging *versus* flexion MR technique of ACL imaging and, *versus* ACL imaging obtained with standard MR protocol of the knee.

**Patients and methods:**

In this prospective study we included 149 patients who were referred to magnetic resonance imaging (MRI) examination due to knee soft tissues trauma during 12 months period. MRI signs of ACL trauma, especially detection of partial tears, number of slices per technique showing the whole ACL, duration of applied additional protocols, and reproducibility of examination were analysed.

**Results:**

Accuracy of standard MRI protocol of the knee comparing to both additional techniques is identical in detection of a complete ACL rupture. Presentations of the partial ruptures of ACL using flexion technique and sagittal-oblique technique were more sensitive (p<0.001) than presentation using standard MR protocol. There was no statistically significant difference between MRI detection of the ruptured ACL between additional techniques (p> 0.65). Sagittal-oblique technique provides a higher number of MRI slices showing the whole course of the ACL and requires a shorter scan time compared to flexion technique (p<0.001).

**Conclusions:**

Both additional techniques (flexion and sagittal-oblique) are just as precise as the standard MR protocol for the evaluation of a complete rupture of the ACL, so they should be used in cases of suspicion of partial rupture of the ACL. Our study showed sagittal-oblique technique was superior, because it did not depend on patient’s ability to exactly repeat the same external rotation if standard MR protocol was used or to repeat exactly the same flexion in flexion MR technique in further MR examinations. Sagittal-oblique technique does not require the patient’s knee to be repositioned, which makes this technique faster. We propose this technique in addition to the standard MR protocol for detection of partial ACL tears.

## Introduction

Anterior cruciate ligament (ACL) injuries occur due to a strong contact or indirect knee trauma, and results in stretching or tearing of non-contractile, elastic soft-tissue articular structures. ACL is the most frequently injured large ligament in the knee.^1^ As injured ACL recovery is very limited long-term consequences are frequent including cartilage loss, secondary meniscal injuries and degenerative changes.

Magnetic resonance imaging (MRI) examination gives precise information about lesions in different organs as well as in the knee.^2^–^5^ It determines the course and length of patient’s treatment. MRI diagnostic protocols and technical abilities have almost completely excluded the use of diagnostic arthroscopy nowadays.

Partial ACL tears occur in 10–43% of all ACL injuries.^5^–^8^ In cases of partial ACL tears conservative treatment is effective in less than 25% in diameter. Larger tears progress into a complete rupture in 50–86% of cases.^8^

Partial ACL tears are difficult to diagnose during physical examination. On the other hand, many studies emphasise insufficiency of standard MRI protocol in diagnosing partial ACL tear.^9^–^11^ There are, predominantly, two approaches in published studies, considering additional MRI examination of the ACL:

- dedicated MRI examination with one’s knee in a slightly flexed position (approximately 17 degrees) and sagittal slices along the ACL, trying to avoid partial averaging with the intercondylar roof in the ACL central and proximal part – flexion technique;^12^–^15^
- dedicated MRI examination consisting of images trough longitudinal course of the ACL obtained with angled coronal and angled sagittal tomograms, the “double-oblique” or sagittal-oblique technique.^16^,^17^

The study goal was to compare sagittal-oblique MR technique *versus* flexion MR technique for ACL imaging with standard knee MR protocol. We analysed diagnostic value of MRI data related to ACL trauma, especially detection of partial tears, number of slices per technique showing the whole ACL, duration of additional protocols, ergonomics and reproducibility of examination.

## Patients and methods

The study included 149 patients (age distribution Table 1) referred to MRI examination in Centre for Diagnostic Radiology in Clinical Centre of Montenegro during 12 months period, due to trauma of knee soft tissue structures. Inclusion criteria were positive history for ACL lesion and/or positive one or two ACL lesion physical tests (the Lachman and anterior drawer test).^6^,^8^,^18^

Radiological criteria for diagnosing ACL lesions obtained by standard MRI protocol and by additional techniques were identical: complete rupture (total discontinuity or an area of increased signal intensity extending completely across the ACL, partial rupture (inhomogeneity and increased signal intensity within the ACL, but with some fibres intact) and normal.^19^,^20^ Partial ACL rupture parameters were: direct presentation of partial rupture of ACL, course of the ligament, presented by “increased curvature of posterior convexity of ACL”^9^,^10^ and intraligamentous hyperintense MRI signal.^21^,^22^

Additionally, the number of slices showing the whole ACL, duration of particular sequences and total time of additional techniques were analyzed for two dedicated ACL imaging techniques.

All patients were examined with standard MRI protocol for the knee followed by sagittal-oblique technique and flexion technique using MR 1.0T scanner (Philips, Intera, Eindenhoven, The Netherlands) with omni gradient 23 mT/m and, software version 11. Standard MRI protocol for the knee consisted of triplanar images using T2 fast-spin echo sequences (repetition time [TR] 3382, echo-time [TE] 100, flip angle 90 degrees), 3 mm slices with 0.3 mm gap and field of view [FOV] of 170 mm, 512x256 matrix. Patient was in supine position with extended knee in external rotation.^23^ After standard MRI protocol, additional sagittal-oblique technique and flexion technique were performed in all patients using same parameters as standard MRI protocol with the exception for slice thickness of 2 mm and 0.2 mm gap.

**FIGURE 1.**
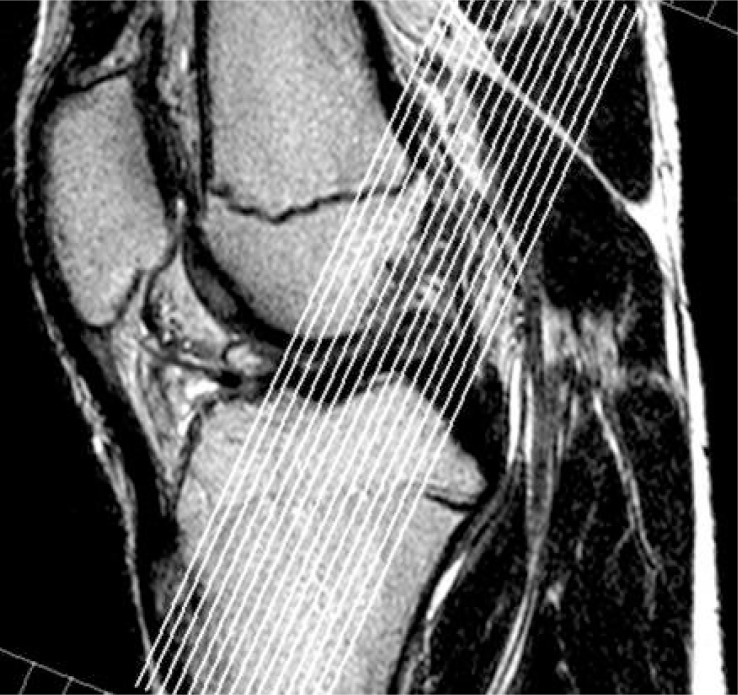
Sagittal image of standard MRI examination as a topogram for planning the paracoronal oblique T2 FSE 2 mm image.

**FIGURE 2.**
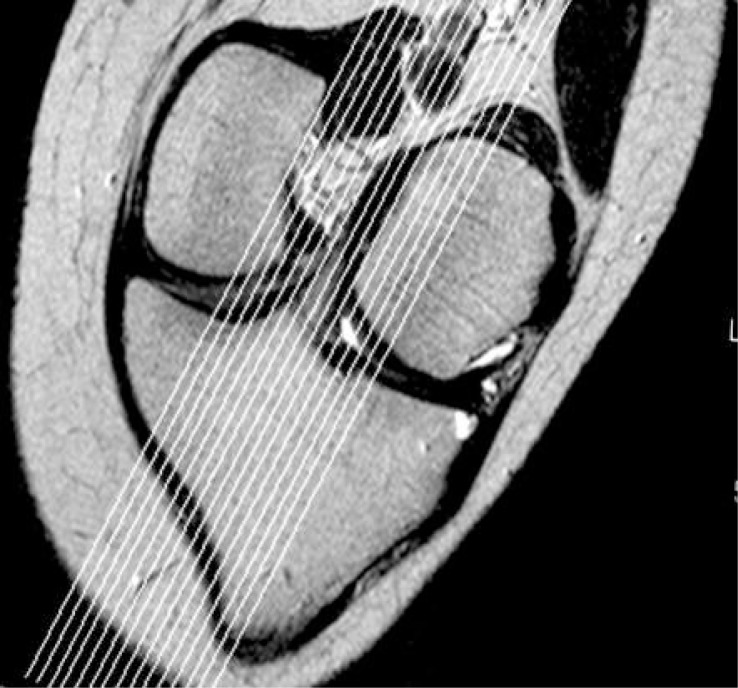
Anterior cruciate ligament (ACL) paracoronal oblique image.

**FIGURE 3.**
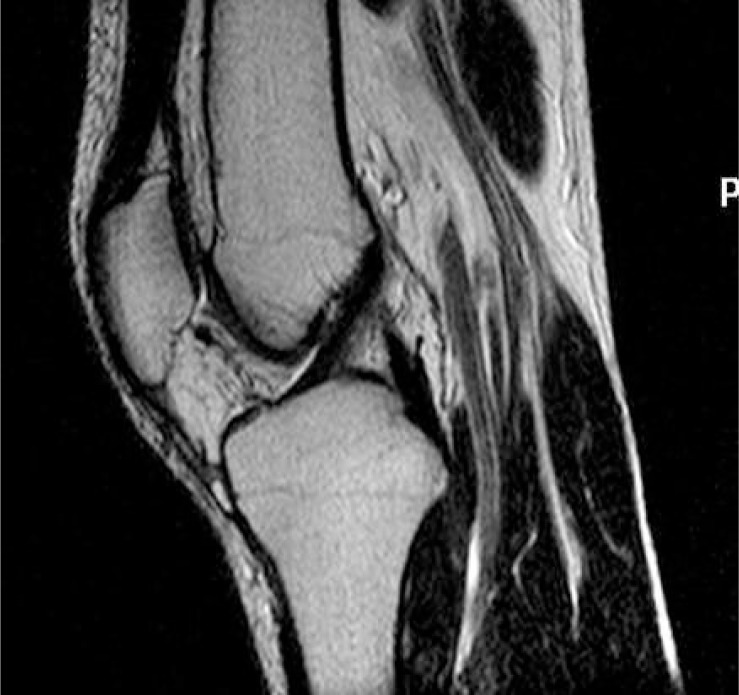
Anterior cruciate ligament (ACL) sagittal-oblique T2 FSE image.

Sagittal tomogram of the knee with the best image of the ACL, obtained with the previously applied standard MRI protocol was used as a topogram for paracoronal oblique sequence that followed the actual course of ACL (Figure 1). Obtained paracoronal oblique image was angled depending on actual anatomy of the patient’s knee (Figure 2). This image, showing the full course of ACL, was used as a topogram for the sagittal-oblique protocol (Figure 3).

**FIGURE 4.**
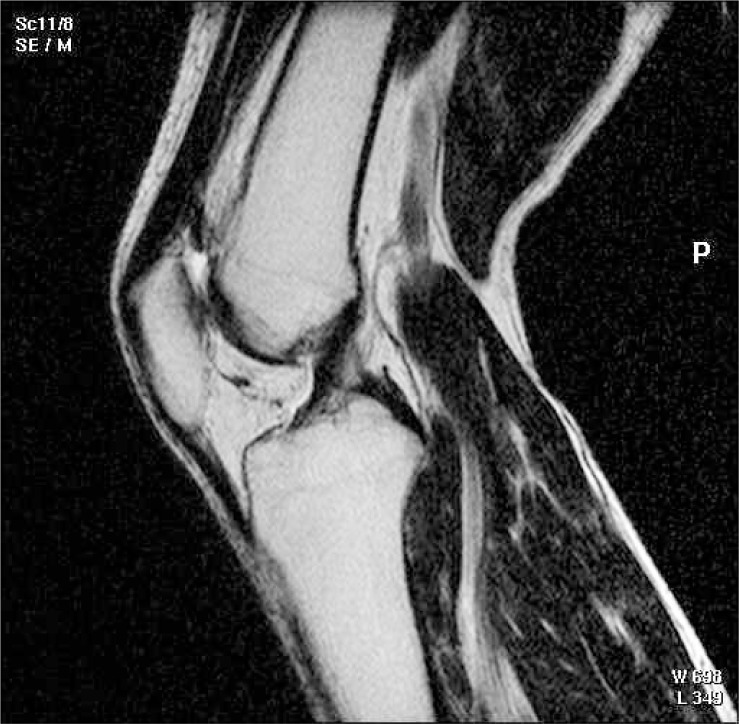
Anterior cruciate ligament (ACL) flexion MRI technique.

After the sagittal-oblique MRI examination, the flexion MRI technique was performed. This technique requires repositioning of patients knee in 17 degree flexion, followed by a sagittal topogram with orientation of slices without angling (Figure 4).

Two independent radiologists analysed the MRI figures of the patients, their history and physical data.

Data collected from the patients that showed a variety of ACL injuries diagnosed by the compared techniques (standard MR protocol, sagittal-oblique MR technique and flexion MR technique) were assessed by a chi-square test. Statistical differences between variants expressed as arithmetic averages and percentages were assessed by the Student’s t-test.

All patients signed the consent of participation in the study. The investigators strictly followed recommendations of the Helsinki Declaration (1964, with later amendments) and of the European Council Convention on Protection of Human Rights in Bio-Medicine (Oviedo 1997).

## Results

All 149 patients (108 males, 41 females) were sent to MRI examination with a clinical diagnosis: internal knee lesion-meniscal or ACL lesion. In 59 patients (39.6%) referring diagnosis was decisive – ACL lesion.

All patients suffered from a knee injury which mechanism pointed to the lesion of internal soft tissue structures and thus potentially to the ACL injury. The anterior drawer test was positive in 94 patients (63.1%), out of them 86 (57.7% of total patients) had positive Lachman test. In the remaining 55 (36.9%) patients, MR examination was performed on the basis of the mechanism of injury (from patient’s history) and clinical suspicion of a violation of the ACL.

Standard knee MR protocol was positive (complete or partial ACL lesion) in 134 patients (89.9%) out of 149 patients. The percentage of complete lesion of ACL using standard MR protocol was 42.3%. The percentage of partial lesion of ACL using standard MR protocol was 47.6% (Table 2).

Both, flexion MRI technique and sagittal-oblique technique showed the same number of complete ACL ruptures, 63 (42.3%), as in standard MR protocol of the knee. The number of diagnosed partial ACL ruptures using flexion technique and sagittal-oblique technique was 86 in both techniques respectively, comparing to 71 ACL ruptures diagnosed by standard MR protocol.

The percentage (47.9%) of direct presentation of the partial rupture of ACL using standard MR protocol was statistically significantly lower (p<0.001) than in flexion technique (77.9%).

Also, higher percentage of direct presentation of partially ruptured ACL of 82.5% using sagittal-oblique technique compared to 47.9% using standard knee MR examination was statistically significant (p<0.001) (Table 3). The percentage of direct presentation of partially ruptured ACL using sagittal-oblique technique in comparison to flexion technique was without statistical significance (p> 0.65).

The site of partial ACL rupture detected by MRI (upper attachment, middle part or lower attachment) using flexion and sagittal-oblique technique showed no statistically significance between these techniques with *p* value less than 0.05 (Table 4).

Number of slices showing the whole course of the ACL and the time of examination comparing flexion and sagittal-oblique technique are presented in Table 5. Sagittal-oblique technique provides a higher number of slices presenting the whole course of ACL. The necessity of repositioning of the patient in order to perform flexion technique reduces reproducibility in possible subsequent examinations. Shorter scan time of 1.9 minutes required for sagittal-oblique technique (1 minute 54 seconds) compared to the duration of flexion technique is statistically significant (p<0.001).

## Discussion

Complete rupture of the ACL does not represent a diagnostic problem for the standard MR protocol of the knee as overall accuracy of the procedure ranges from 86% to 100%.^10^,^11^,^20^,^21^,^24^–^31^ Lower accuracy of standard MR protocol for partial rupture of ACL can be improved by using additional, dedicated MR techniques, described in recent literature as “standard orthogonal sequences plus oblique axial intermediate weighted imaging”.^32^ We tested two additional MR techniques for diagnosing partial ACL tear and confirmed them to be more sensitive (p<0.001) than standard MR protocol, with no statistical difference between them.

Sagittal-oblique technique clearly shows partial rupture because its’ double angulation follows the specific course of the patient’s ligament, due to approximate orientation of the external rotation of the foot. The advantages of this technique were described in MR studies of the knee after ligamen-toplasty.^28^,^33^–^35^

In flexion technique, the flexed knee position avoids partial volume effect of intercondylar fossa of the ACL, which is one of the major diagnostic problems in diagnosing partial ACL rupture using standard MR protocol.^7^,^30^,^36^,^37^ In addition, un-streched ligament in the knee in flexion is wider and thus can be precisely analysed.^14^

We shoved that both additional MR techniques reveal partial rupture of ACL from the “grey zone” of indirect signs such as course angulations of ACL, hyperintense MR signal, unclear contours of the ligament with the characteristics of fluid, bleeding and/or fibrosis (depending on the time between trauma and MRI examination).^7^,^11^,^21^,^26^–^29^,^34^

Intraligamentous hyperintense signal on T2 sequences, as a sign of fluid accumulation between the ACL-bundles due to fibre microruptures, found on standard MR procedure and additional techniques, showed the same level of sensitivity. Flexion technique seemed to be slightly more precise in detecting an intraligamentous hyperintense signal than standard MR protocol and sagittal-oblique technique because the flexion of the knee during examination results in shortening and widening of ACL, making the fluid in the ligament thicker and thus more available for detection.

In assessment of the location of ACL partial rupture (upper attachment, middle part, lower attachment) both flexion technique and sagittal-oblique technique showed the same results, confirming lower attachment as the most common site of ACL partial rupture.^11^,^35^

In our study we found the statistically significant difference between standard procedure and both, flexion technique and sagittal-oblique technique regarding the number of MR slices that showed the whole course of ACL. Sagittal-oblique technique offers a larger number of MR tomograms showing the whole ACL, due to angulations in two planes with two phases of sagittal and paracoronal tomograms which present the true ligament axis of the patient’s knee, making the ACL clearly visible. This technique does not require repositioning of the patient as flexion technique, thus the reproducibility of this method is easier and we have recommended it in patients with expected re-examinations. On the other hand, this technique is more ergonomic and faster because there is no need for patient knee repositioning and for a new scout.

Clinical parameters (patient’s history, clinical examination) are not the subject of this study, but in our study we found low percentage of precise referring diagnoses (only 39.6%), many unclear referring diagnosis (internal knee lesion) and various time between knee trauma and MR examination.

Lachman test was positive in fewer patients than the anterior drawer test, mostly in patients with strong quadriceps muscles as a result of greater physical effort that had to be applied by physician while performing anterior drawer test. This statement differs from the literature^25^, probably due to a larger number of partial ruptures of the ACL in our study that are difficult to diagnose with physical examination.^8^ Our study emphasises the role of MRI examination, especially for partial ACL lesions and confirms literature statements.^5^,^7^,^11^,^21^,^26^–^28^

The limitation of our study is the lack of arthroscopic procedures (as a “gold standard”) after MR examinations. Arthroscopies were not performed in patients with clinical suspicion of isolated ACL injury, or minor lesions of the knee (mostly referred as “internal knee trauma”). If isolated partial tear of ACL was diagnosed on MR, patients were treated conservatively with physiotherapy and rehabilitation therapy. These approaches reduce the risk of invasive diagnostics and also the coast of patient’s treatment.^19^

## Figures and Tables

**TABLE 1.** Patient’s distribution by age groups

**Age groups**	**No of patients**	**%**
<10 years	4	2.7%
10–20 years	17	11.4%
20–30 years	49	32.9%
30–40 years	71	47.6%
40–50 years	8	5.4%
Total	149	100%

**TABLE 2.** Standard knee MR protocol, flexion MR technique and sagittal-oblique technique findings of anterior cruciate ligament (ACL)

**Anterior cruciate ligament (ACL)**	**Standard MR protocol**	**Flexion technique**	**Sagittal-oblique technique**
Complete rupture	63 (42.3%)	63 (42.3%)	63 (42.3%)
Partial rupture	71 (47.6%)	86 (57.7%)	86 (57.7%)
Normal	15 (10.1%)	0	0
Total	149 (100%)	149 (100%)	149 (100%)

**TABLE 3.** Standard knee MR protocol, flexion MR technique and sagittal-oblique MR technique findings of partial rupture of anterior cruciate ligament (ACL)

**ACL partial rupture**	**Standard MR protocol**	**Flexion technique**	**Sagittal-oblique technique**
Direct presentation	34 (47.9%)	67 (77.9%)	71 (82.5%)
Course of ACL	22 (31%)	0	0
Hyperintens signal	15 (21.1%)	19 (22.1%)	15 (17.5%)
Total	71 (100%)	86 (100%)	86 (100%)

**TABLE 4.** Flexion MR technique and sagittal-oblique MR technique findings of the position of partial rupture of anterior cruciate ligament (ACL)

**Position of the partial rupture of ACL**	**Flexion technique**	**Sagittal-oblique technique**
Upper attachment	30 (34.9%)	29 (33.7%)
Middle part	20 (23.2%)	16 (18.6%)
Lower attachment	36 (41.9%)	41 (47.7%)
Total	86 (100%)	86 (100%)

**TABLE 5.** Differences between flexion MR technique and sagittal-oblique MR technique in terms of number of tomograms that show the whole course of the anterior cruciate ligament (ACL) and the total duration of examination

	**Flexion technique**	**Sagittal-oblique technique**
**Number of tomograms showing all coarse of ACL**	2.7	3.8
Reposition of the patient	5.1 min	0
Scout / T2, 2 mm	0.5 min	3.9 min
T2, 2 mm / T2, 2 mm	4.2 min	4 min
Total time	9.8 min	7.9 min
